# Coloring Effects of Disinfectants on Pure or Nano-TiO_2_-Incorporated Maxillofacial Silicone Prostheses

**DOI:** 10.3390/ma16165580

**Published:** 2023-08-11

**Authors:** Pinar Cevik

**Affiliations:** 1 Department of Prosthodontics, Faculty of Dentistry, Gazi University, 06490 Ankara, Türkiye; pinarcevik@gazi.edu.tr or pinar.cevik@uth.tmc.edu; 2 Department of General Practice and Dental Public Health, School of Dentistry, The University of Texas Health Science Center at Houston, Houston, TX 77054, USA; 3 Houston Center for Biomaterials and Biomimetics, Houston, TX 77054, USA

**Keywords:** aging, color differences, disinfectants, maxillofacial prosthesis, silicone elastomers

## Abstract

Silicone elastomers play a crucial role in the field of maxillofacial prosthodontics. To maintain optimal hygiene, various disinfectants have been reported to clean silicone prostheses. Nevertheless, when selecting a disinfectant, it is important to consider not only its antimicrobial efficacy, but also its compatibility with the materials, to minimize any potential impact on the physical properties of the material surfaces. The coloring effect of such disinfectants on different types of silicone is of interest. A total of 144 silicone specimens (72 pure silicones, 72 nano-TiO_2_-incorporated silicones, from A-2000 and A-2006 silicones) were fabricated in this study. The spectrophotometric analysis was carried out, and the initial CIE L*a*b* color values were measured prior to disinfection. Specimens in each silicone group (with or without nano-TiO_2_) were subjected to a 30-h disinfection period simulating 1 year of disinfection with the following disinfectants: Control (tap water), 0.2% chlorhexidine gluconate, 4% chlorhexidine gluconate, 1% NaOCl, neutral soap, and effervescent. After the second color values were recorded, the color change (∆E*) was calculated. Significant differences were observed among the disinfectants for both the A-2000 and A-2006 silicone groups. Nano-TiO_2_ did not show a color protection effect in A-2000 silicone. In contrast, nano-TiO_2_ incorporation provided color protection against CHG 0.2%, CHG 4%, and NaOCl in A-2006 silicone. Most of the disinfectants did not show acceptable color stability over time. In pure A-2000 silicone, except for 0.2% chlorhexidine, all disinfectant groups demonstrated a color change within the acceptability threshold of 50:50% (∆E* = 3.0). On the other hand, in nano-TiO_2_-incorporated A-2006 silicone, only 0.2% and 4% chlorhexidine demonstrated an acceptable color change. Overall, chlorhexidine could be used as a suitable disinfectant in maxillofacial silicone prostheses.

## 1. Introduction

The restoration of facial aesthetics and improvement of quality of life are primary goals for patients with facial deformities requiring maxillofacial prostheses [1]. Despite significant advancements in plastic and reconstructive applications, the demand for maxillofacial prosthetics remains constant, especially for patients with unrestorable defects. To address this need, recent developments in maxillofacial prosthodontics have embraced the integration of 3D printing technologies in both the design and fabrication of prostheses [2,3,4]. The physical and chemical properties of silicone elastomers, which are commonly used in facial prostheses, are influenced by factors such as crosslinking within their structure and the type and concentration of fillers in the elastomer network, as well as thermal initiators, other additives, polymerization time, and temperature [4,5,6]. These factors collectively impact the strength and durability of the silicone material, thus affecting its overall lifespan [2,5]. Recently, most studies have aimed to identify the most biocompatible materials with the longest lifespan for use in maxillofacial prostheses [6,7,8]. In this regard, there has been a growing interest in improving the mechanical or physical properties of elastomers by incorporating nano-oxide particles, such as ZnO, TiO_2_, and CeO_2_, as fillers in silicone-based elastomers [1,9,10]. It was reported that the use of a reflection spectrophotometer as a method has been widely utilized for assessing the color stability of maxillofacial elastomers. On the other hand, colorimeters also provide color measurements in the CIE L*a*b* format, which allows for mathematical analysis and facilitates the comparison of color parameters between different objects [11].

Studies have reported the colonization of complex microbial biofilms in maxillofacial prostheses. This colonization can lead to various issues, such as skin irritation and disuse of the prosthesis, ultimately resulting in the need for prosthesis replacement [12,13]. Cleaning of silicone prostheses can be achieved through various methods, including mechanical cleaning using manual brushing or hand washing with neutral soap, as well as chemical cleaning with nontoxic disinfectants [14]. Conventional cleaning processes such as scrubbing with soap have the potential to degrade the surface of the prosthesis, which can result in colonization by microbes. Moreover, mechanical cleaning methods may contribute to the deterioration of the silicone material and may not effectively eliminate all accumulated bacterial colonies [1,13,15]. In addition, repeated mechanical scrubbing should be avoided, as it may lead to the dissolution and removal of pigments present on the external surface [16]. In contrast, chemical disinfectants offer a viable solution, as they can effectively reduce the risk of infection without causing any adverse alterations to the physical properties of the silicone [16,17]. Based on the current literature, chlorhexidine at various concentrations seems to be the gold standard for disinfecting maxillofacial prostheses [13,14,18]. In recent years, several studies have been conducted to investigate the effects of various disinfectants on the color stability of maxillofacial silicone elastomers [1,13,14,18,19]. Differences observed in studies investigating the color and mechanical properties of maxillofacial silicones after disinfection can be attributed to variations in study design and the materials used. Furthermore, when selecting a disinfectant, consideration should be given to its antimicrobial properties, compatibility, and inertness, in order to minimize any adverse impact on the physical properties of the material’s surface and preserve them to the greatest extent possible.

The specific chemical composition and processing methods of various silicone elastomers play a critical role in influencing their physical and mechanical properties. Due to the distinct matrix structure found in different silicones [8,20,21], each silicone can exhibit varied physical properties. Additionally, the presence of different chemical bonds in each silicone group may contribute to varying color stability results. Consequently, conducting a study that tests disinfecting agents and their physical effects using different types of silicone is essential. Understanding potential differences between materials and disinfectants can provide valuable insights into their interactions and implications for practical applications. By investigating these aspects, we can gain a deeper understanding of the relationship between silicone types and disinfectant effects, which will be beneficial for future research and clinical use.

Pigments are known to provide color protection in specific scenarios [10,20], and their presence in silicone elastomers can influence color stability. However, to achieve better standardization and facilitate data interpretation across various elastomer types, it is essential to evaluate the effects of disinfectants and nano-oxides on unpigmented silicone elastomers as well. In this regard, the evaluation of disinfectants and nano-oxides on unpigmented silicone elastomers serves the purpose of achieving better standardization and facilitating data interpretation across various elastomer types. Consequently, utilizing unpigmented silicones in such studies can lead to a better understanding of the effects of these treatments. In this regard, the first part of our study aimed to evaluate the antimicrobial properties of incorporating nano-oxides and different disinfectants in unpigmented maxillofacial silicone elastomers [18]. This investigation helped to establish the efficacy of these treatments in providing antimicrobial protection to silicone, which is crucial for its clinical application in maxillofacial prosthetics. The second part focused on examining how the incorporation of these disinfectants and nano-oxides influenced the color stability of different types of maxillofacial silicone.

Therefore, the objective of this study was to investigate the potential effects of nano-oxide incorporation and disinfectant type on the color stability of different types of maxillofacial silicone. By utilizing unpigmented silicone elastomers and considering various materials, this research aims to contribute valuable insights into the field of maxillofacial prosthetics and enhance our understanding of how these treatments can impact color stability in different clinical scenarios. The null hypotheses of this study were that (1) the disinfectants used to clean the silicone elastomers would not have any effect on the color stability of the silicone elastomers, and that (2) nano-TiO_2_ would not affect the color stability of different silicone elastomers after being subjected to disinfection for 30 h.

## 2. Materials and Methods

**Figure 1:**
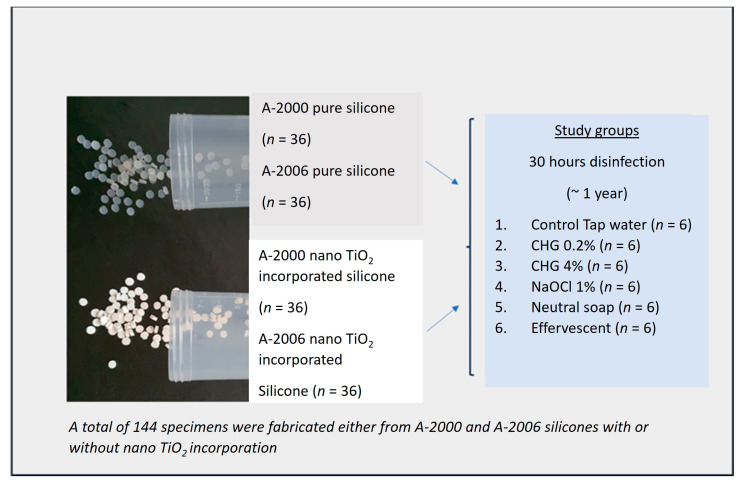
Study groups in a schematic design.

The aim of this study was to evaluate the color stability of 2 different platinum-type RTV (room-temperature-vulcanized) silicone elastomers, A-2000 and A-2006 (Factor II Inc., Pinetop-Lakeside, AZ, USA), and 2 subgroups within each elastomer type, consisting of specimens with either pure silicone or nano-TiO_2_ incorporation (Figure 1).

The mixing process involved combining part A and part B of each silicone in a glass container at a 1:1 ratio. In order to prevent the formation of air bubbles, 3 drops of thixotropic agent were added to each 10 g silicone mixture for the pure silicone groups. Another mixture was created using the same silicone types and procedures, with the addition of 30 nm nano-TiO_2_ particles (10% by volume) (IMICRYL Inc., Konya, Turkiye). A punch was used to cut the testing specimens from 2 mm thick flat silicone specimens. A total of 144 rounded specimens with 2 mm thickness were fabricated from either A-2000 or A-2006 silicones, with or without nano-TiO_2_ incorporation. The polymerization process was conducted at 60 °C for 6 min using a hydraulic press (HD80; Hidroliksan Halim Usta Pres San. Tic. Ltd., Konya, Turkiye), following the procedure described previously [15]. Prior to conducting the disinfection procedure, a spectrophotometric analysis was carried out under the same gray background, and the initial CIE L*a*b* color values were measured as described by Kiat-amnuay et al. [11]. Then, specimens from each subgroup of silicone were divided into 6 groups (*n* = 6) according to the cleaning solutions used: control—tap water (no disinfection), 0.2% chlorhexidine gluconate (0.2% CHG), 4% chlorhexidine gluconate (4% CHG), 1% sodium hypochlorite (1% NaOCl), neutral soap (natural soap), and alkaline peroxide effervescent denture-cleaning tablets (effervescent). The silicone specimens underwent disinfection using various disinfecting agents, with each agent applied for a duration of 30 h. This disinfection period was chosen to simulate approximately 1 year of clinical use. The rationale behind this choice is that 30 h of disinfection within a 1-year period can be considered equivalent to a 3–8 min daily treatment, as supported by previous studies [19,21,22]. This approach ensures that the specimens experience a realistic and relevant disinfection process, reflecting potential long-term usage conditions. After completing the disinfection process, the second set of CIE L*a*b* color values was recorded. The color change (∆E*) was calculated using the following formula: ∆E* = [(∆L*)2 + (∆a*)2 + (∆b*)2]1/2, where ∆L* is the change in L* between the interval of interest and baseline, ∆a* is the change in a* between the interval of interest and baseline, and ∆b* is the change in b* between the interval of interest and baseline. This calculation allows for the quantification of color changes in the specimens.

Statistical analysis of the data was performed using IBM SPSS Statistics software, version 29.0 (IBM Corp., Chicago, IL, USA). Descriptive statistics, including mean values, standard deviations, medians, minimum and maximum values, and interquartile ranges (IQRs), were calculated for the dataset. The Kruskal–Wallis test was used to evaluate the effects of the disinfectants. The Mann–Whitney U test was used to analyze the differences between the pure and TiO_2_-incorporated groups in both A-2000 and A-2006 silicones with each disinfectant. To account for multiple comparisons among disinfectant groups in each of the TiO_2_ or pure A-2000 and A-2006 silicones, significance values were adjusted using the Bonferroni correction in this study. The significance level was set to *p* < 0.05. To prevent confusion, this study only reports median values with IQRs, since the analysis conducted was nonparametric.

## 3. Results

Significant differences were observed among the disinfectants for both the A-2000 and A-2006 silicone groups (*p* < 0.05). Table 1 and Table 2 show the summary of the independent-samples Kruskal–Wallis test results regarding the differences between disinfectant groups in each subgroup (with or without TiO_2_) and group of silicones. According to Table 1 and Table 2, there were statistical differences among the disinfectants in each subgroup of silicones (pure or nano-TiO_2_-incorporated) for both A-2000 (Table 1) and A-2006 silicones (Table 2) (*p* < 0.05).

Table 1 and Table 2 present the median values with interquartile ranges (IQRs) for each group in this study. Based on the results in Table 1 and Table 2, there were statistically significant differences between the disinfectant groups, whether TiO_2_-incorporated or pure, within the A-2000 and A-2006 silicone groups. On the other hand, care was taken to interpret the data regarding the 50:50% acceptability threshold (∆E* = 3.0) for the color of maxillofacial silicone prostheses in this study [23,24]. 

Table 1 shows the color change of TiO_2_-incorporated and pure A-2000 silicone elastomers after being subjected to disinfection. Significant differences between disinfectants were observed in nano-TiO_2_-incorporated and pure A-2000 silicones (*p* < 0.05). Regarding this, in nano-TiO_2_-incorporated A-2000 silicone, the ΔE* value of CHG 0.2% (ΔE* = 3.94) was statistically lower than that of the tap-water group (ΔE* = 15.41). There were no statistical differences among the other groups in the nano-TiO_2_-incorporated A-2000 silicone (*p* > 0.05). In pure A-2000 silicone, statistical differences were found between the disinfectants (*p* < 0.05). The ΔE* values of neutral soap (ΔE* = 1.15) and NaOCl (ΔE* = 1.11) were found to be statistically lower than those of CHG 0.2% (ΔE* = 5.01) and tap water (ΔE* = 4.76). There were no statistical differences between the groups CHG 0.2%, CHG 4%, effervescent, and tap water (*p* > 0.05). 

Table 1 demonstrates that pure A-2000 silicone exhibited statistically lower color change than nano-TiO_2_-incorporated A-2000 silicone in the neutral soap, effervescent, CHG 0.2%, and tap-water groups (*p* = 0.02, *p* = 0.04, *p* = 0.02, and *p* = 0.02, respectively). 

Table 2 shows the color change of TiO_2_-incorporated and pure A-2006 silicone elastomers following disinfection. Significant differences were observed between the disinfectants in both nano-TiO_2_-incorporated and pure A-2006 silicones (*p* < 0.05). In nano-TiO_2_-incorporated A-2006 silicone, the color change of CHG 0.2% (ΔE* = 2.32) or CHG 4% (ΔE* = 2.18) was statistically lower than that of tap water (ΔE* = 8.04). In pure A-2006 silicone, the ΔE* values of neutral soap and tap water were statistically lower than that of NaOCl. However, no statistical differences were found between neutral soap, effervescent, CHG 0.2%, and CHG 4%. 

Table 2 indicates that in the CHG 0.2%, CHG 4%, and NaOCl groups, nano-TiO_2_-incorporated A-2006 silicone exhibited statistically lower ΔE* values than pure A-2006 silicone (*p* = 0.015, *p* = 0.02, and *p* = 0.02, respectively).

Control (tap water) specimens had a color change of 4.76 ∆E* in A-2000 and 2.12 ∆E* in A-2006 silicone elastomers. However, upon TiO_2_ incorporation, the color change increased to 15.41 ∆E* in A-2000 and 8.04 ∆E* in A-2006 silicones. Notably, the incorporation of nano-TiO_2_ led to a statistically higher color change only in A-2000 silicone when compared with the pure A-2006 silicone in control (tap water) specimens in this study.

Among the disinfectant groups, the color changes of CHG 0.2% (∆E* = 2.32) and CHG 4% (∆E* = 2.18) were below the acceptability threshold of ∆E* = 3.0 in the TiO_2_-incorporated A-2006 silicone elastomer. Additionally, TiO_2_ incorporation resulted in a lower color change as compared with pure silicone for A-2006 silicone elastomers in all disinfectant groups except for the control (tap water).

In the A-2000 silicone elastomer, TiO_2_ incorporation resulted in increased color change, as compared with pure silicone, in the neutral soap, effervescent, CHG 4%, and tap-water (control) groups. Additionally, the color changes of the neutral soap (∆E* = 1.15), effervescent (∆E* = 2.24), CHG 4% (∆E* = 2.31), and NaOCl (∆E* = 1.11) groups were within the acceptability threshold of ∆E* = 3.0 in the pure A-2000 silicone group, except for the CHG 0.2% (∆E* = 5.01) and control (tap water) (∆E* = 4.76) groups. 

**Figure 2:**
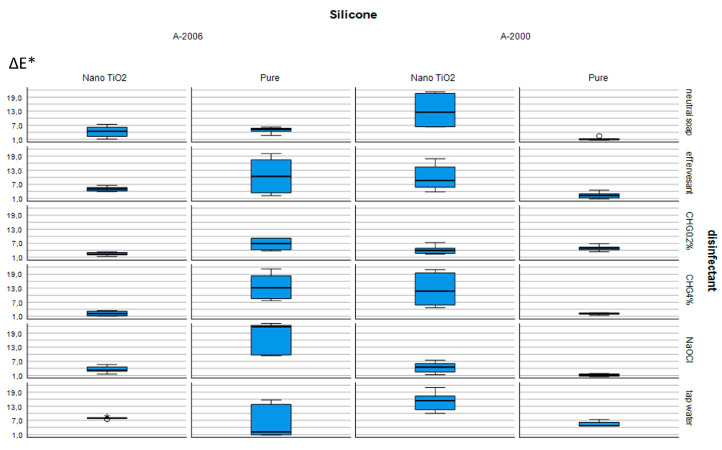
Median ΔE* values with IQRs for all groups in this study.

As shown in Table 1, CHG 0.2% showed a statistically lower color change than that of the control (tap water) in nano-TiO_2_-incorporated A-2000 silicone (*p* < 0.05). In addition, NaOCl showed a statistically lower color change than that of the control (tap water) in pure A-2000 silicone (*p* < 0.05) (Table 1). There were no statistical differences among the other study groups in pure A-2000 silicone. In contrast, NaOCl showed a statistically higher color change than that of the control (tap water) in pure A-2006 silicone (*p* < 0.05) (Table 2). As shown in Table 2, both CHG 0.2% and CHG 4% indicated statistically lower color changes than that of the control (tap water) in nano-TiO_2_-incorporated A-2006 silicone (*p* < 0.05). Figure 2 also shows ΔE* values with IQRs as a graphical representation for all of the groups included in the study.

## 4. Discussion

The null hypotheses of this study were rejected, as different disinfecting agents had different effects on the color stability of the silicone elastomers. Additionally, the incorporation of nano-TiO_2_ statistically affected the color change in A-2006 silicone.

Significant differences were observed among the disinfectant groups in this study. On the other hand, it is important to interpret the data obtained in color studies based on the 50:50% acceptability threshold (∆E* = 3.0) [23,24]. Therefore, both statistical differences between the groups and acceptability thresholds were taken into consideration while interpreting the data in this study.

Effervescent tablets, containing peroxides, are known to release oxygen, which aids in the removal of substances and stains accumulated on the surface. However, it has been observed that the release of oxygen can cause color changes in prostheses, particularly in those with extrinsic pigments [25,26]. In our study, only the pure A-2000 silicone group treated with effervescent tablets demonstrated an acceptable color change (∆E* = 2.24). Furthermore, it was observed that the addition of nano-TiO_2_ in silicone treated with effervescent tablets led to a higher degree of color change compared with the pure silicone groups, in both silicone types. This could result from the oxygen mechanism of the chemical agent reacting with the nano-oxides inside the silicone specimens [27]. It is possible for the oxygen mechanism of an effervescent agent to react with nano-oxides inside the silicone, resulting in decreased color stability in nano-TiO_2_ specimens.

Color change may occur in the event of chemical agent adsorption or absorption. CHG has been reported to rapidly and differently adsorb to anatase and rutile TiO_2_ [28]. The findings of this study support the idea that specific interactions may occur between chlorhexidine gluconate (CHG) and titanium dioxide (TiO_2_). In A-2006 silicone, it can be inferred that nano-TiO_2_ interacted with CHG, leading to color protection over time. However, this interaction was not observed in A-2000 silicone, which could be attributed to the differences in the composition and additives of A-2000 and A-2006 silicones. Different silicones can display varying physical properties due to their distinct matrix structures and chemical bonds. The overall results can be influenced by various factors, including the concentration and form of TiO_2_, the pH and temperature of the environment, and the presence of other chemicals or additives. Analyzing specific data in future multidisciplinary studies will help uncover the materials’ exact mechanisms, considering factors like TiO_2_ concentration, pH, temperature, and the presence of other chemicals or additives. 

An unexpected outcome was that the nano-titanium-dioxide was not as stable as assumed in the A-2006 control (tap water) group. Only the control (tap water) group exhibited an acceptable color change in the pure A-2006 silicone group. In contrast, all of the disinfectants resulted in a coloring effect in the pure A-2006 silicone when compared with the control (tap water). However, when nano-TiO_2_ was incorporated, it statistically decreased the color change in the other disinfectant groups for A-2006 silicone. An interesting observation was made in the control (tap water) group, where nano-TiO_2_ exhibited an antagonistic effect. These results suggest that nano-TiO_2_ alone may not provide a color protection effect. However, it appears that a synergistic effect occurs in the presence of a disinfectant, influencing the color change of the A-2006 silicone elastomer. Furthermore, although there has been some research evaluating the color stability of A-2000 silicone, no study has evaluated the color stability of A-2006 silicone maxillofacial elastomers. Therefore, these results could not be compared with those of other studies. However, the data in this study support the idea that color degradation of A-2006 silicone can be protected against by TiO_2_ incorporation when disinfecting with CHG agents over time. These findings also highlight the importance of considering the specific silicone type and its composition when evaluating the impact of nano-TiO_2_ incorporation on color stability. 

Based on the findings of this study, it can be concluded that CHG 0.2% and 4%, as well as NaOCl, demonstrated a positive interaction with nano-TiO_2_ in A-2006 silicone, resulting in a color-protective effect over time that remained within the acceptable threshold. However, in the case of A-2000 silicones, there was no statistically significant difference between pure and nano-TiO_2_-incorporated silicones in the CHG 0.2% and NaOCl groups. Notably, only CHG 0.2% showed a significantly lower color change compared to the control (tap water) group in nano-TiO_2_-incorporated A-2000 silicone. This result aligns with those of a previous study that reported color stability in pigmented A-2000 silicone when treated with CHG 0.2% after accelerated aging [22]. 

The results of this study show that the incorporation of nano-TiO_2_ had varying effects on different types of silicones. Despite both silicones being of the platinum type and from the same manufacturer, their hardness values differed due to the presence of different additives in the matrix. It can be inferred that nano-oxides may influence the color stability of various maxillofacial elastomers in different ways.

The color-fading effect of NaOCl has been reported in various studies [1,7,22]. The results of the current study support the idea that NaOCl affects different silicones in various ways. Specifically, in A-2000 silicone, NaOCl had a negative impact on the color stability of nano-TiO_2_-incorporated silicone as compared with pure A-2000 silicone. Although the difference between nano-TiO_2_-incorporated and pure silicones was not statistically significant in A-2000 silicones disinfected with NaOCl, the color change in the nano-TiO_2_ group was above the acceptability threshold. However, for A-2006 silicone, NaOCl resulted in a statistically lower color change in nano-TiO_2_-incorporated A-2006 silicone compared to pure A-2006 silicone. In this study, TiO_2_ incorporation exhibited a color protection effect against NaOCl-induced fading in A-2006 silicone. Additionally, the color change of the nano-TiO_2_-incorporated A-2006 silicone elastomer (ΔE* = 3.19) was found to be slightly above the acceptability threshold.

Furthermore, in pure A-2006 silicone elastomer, NaOCl disinfection caused a statistically higher color change as compared with the control (tap water) in pure A-2006 silicone (*p* < 0.05). This implies that NaOCl changed the color of the unpigmented A-2006 silicone severely. These findings highlight the need for careful consideration when recommending NaOCl as a cleaning agent for different types of silicone elastomers. 

Considering that predominantly chemical disinfectants were employed in this study, it is important to emphasize that the specific conditions, concentrations, and interactions between the chemical agents and nano-TiO_2_ may have significantly impacted the outcomes. Thus, a comprehensive understanding of the chemical and physical properties of the materials involved is essential for accurately predicting the effects.

The use of unpigmented silicone in this study may be considered a limitation. However, incorporating pigmented silicone would have introduced a significant number of additional parameters, potentially complicating the interpretation of the results. It has been reported that pigments can have a color protection effect in certain scenarios [11]. Additionally, different combinations of products, such as silicones, pigments, and disinfecting agents, may interact chemically, altering material properties and leading to color changes. Future research should consider specific combinations of pigments and silicones to investigate these effects further. Despite this limitation, excluding pigmented silicones from the study was intentional to maintain standardization and focus on evaluating the direct effects of the disinfectants and nano-oxide incorporation, without the confounding influence of pigments. In addition, this study aimed to minimize confusion and ensure clarity in interpreting the findings. Future studies could investigate the effects of these disinfectants and nano-oxide particles on silicone with a single type of pigment, providing further insights into their interactions and implications.

Within the limitations of this study, future research should include pigmented silicone elastomers to conduct a direct interpretation of whether nano-oxide incorporation with pigments affects the color stability of pigmented silicone elastomers under different conditions. Increased number of studies on recently used maxillofacial silicones would make for better comparisons in terms of effect of such disinfectants. The findings of this study, along with other studies, support the idea that cleaning the prosthesis under tap water is not recommended. Clinicians should consider multiple factors, including antimicrobial efficacy, hardness, and color of silicone elastomers, when recommending a suitable cleaning method to patients. 

## 5. Conclusions

Based on the findings of this study, we can conclude the following:

1. Incorporation of 10% nano-TiO_2_ did not show a color protection effect in A-2000 silicone.
2. All disinfectants had a coloring effect in the pure A-2006 silicone elastomer. However, nano-TiO_2_ incorporation provided color protection against CHG 0.2%, CHG 4%, and NaOCl colorings in A-2006 silicone.
3. Nano-TiO_2_ incorporation yielded different results in each silicone type. Specifically, CHG 4%, NaOCl, effervescent, and neutral soap resulted in acceptable color changes in pure A-2000 silicone, while nano-TiO_2_ incorporation did not offer any color protection in A-2000 silicone.
4. Overall, CHG could be considered to be a suitable disinfectant for use on maxillofacial silicones. Future research should prioritize supporting in vitro studies on the clinical outcomes by reporting the real patients’ results.
5. These conclusions provide valuable insights into the impact of nano-TiO_2_ incorporation and different disinfectants on color stability in various silicone elastomers, contributing to the advancement of knowledge in the field of maxillofacial prosthodontics.

## Figures and Tables

**Table 1:** ∆E* color change in each disinfectant for pure and TiO_2_-incorporated A-2000 silicones.

A-2000			
Disinfectants	Nano-TiO_2_ Median (IQR)	Pure Median (IQR)	*p*-Value
Neutral soap	12.57 (6.43–20.72) ^ab^	1.15 (0.77–1.59) ^a^	**0.002**
Effervescent	8.55 (5.20–15.28) ^ab^	2.24 (1.05–3.27) ^ab^	**0.004**
CHG 0.2%	3.94 (2.67–5.49) ^b^	5.01 (3.99–5.73) ^b^	0.310
CHG 4%	11.78 (5.60–19.86) ^ab^	2.31 (1.77–2.43) ^ab^	**0.002**
NaOCl	4.52 (2.14–6.41) ^ab^	1.11 (0.64–1.64) ^a^	0.080
Control–tap water	15.41 (11.19–18.30) ^a^	4.76 (4.61–6.51) ^b^	**0.002**

Note: Different superscript letters indicate significant statistical differences (*p* < 0.05) between groups within each column for each disinfectant; bolded *p*-values indicate the differences within each row for nano-TiO_2_ or pure A-2000 silicones (*p* < 0.05).

**Table 2:** ∆E* color change in each disinfectant for pure and TiO_2_-incorporated A-2006 silicones.

A-2006			
Disinfectants	Nano-TiO_2_ Median (IQR)	Pure Median (IQR)	*p*-Value
Neutral soap	4.44 (1.98–6.50) ^ab^	5.29 (4.03–5.91) ^b^	1.000
Effervescent	4.97 (4.11–5.85) ^ab^	10.37 (3.09–18.12) ^ab^	0.589
CHG 0.2%	2.32 (1.76–3.17) ^a^	6.91 (4.16–9.15) ^ab^	**0.015**
CHG 4%	2.18 (1.21–3.24) ^a^	13.20 (8.39–18.98) ^ab^	**0.002**
NaOCl	3.19 (2.43–4.85) ^ab^	21.74 (9.64–23.70) ^a^	**0.002**
Control—tap water	8.04 (7.85–8.26) ^b^	2.12 (0.85–14.27) ^b^	0.394

Note: Different superscript letters indicate significant statistical differences (*p* < 0.05) between groups within each column for each disinfectant; bolded *p*-values indicate the differences within each row for nano-TiO_2_ or pure A-2006 silicones (*p* < 0.05).

## Data Availability

The data presented in this study can be obtained from the corresponding author.
